# Nationwide incidence of anterior cruciate ligament reconstruction in higher-level athletes in Sweden: a cohort study from the Swedish National Knee Ligament Registry linked to six sports organisations

**DOI:** 10.1136/bjsports-2024-108343

**Published:** 2024-11-21

**Authors:** Daniel Castellanos Dolk, Henrik Hedevik, Helena Stigson, Per Wretenberg, Joanna Kvist, Anders Stålman

**Affiliations:** 1 Department of Molecular Medicine and Surgery, Stockholm Sports Trauma Research Center, Karolinska Institutet, Stockholm, Sweden; 2 Department of Orthopedic Surgery, Capio Specialistvård Motala, Motala, Sweden; 3 Unit of Physiotherapy, Department of Health, Medicine and Caring Sciences, Linköping University, Linköping, Sweden; 4 Division of Insurance Medicine, Department of Clinical Neuroscience, Karolinska Institutet, Stockholm, Sweden; 5 Folksam Research, Folksam Insurance Group Stockholm, Stockholm, Sweden; 6 Örebro University School of Medical Sciences, Örebro, Sweden; 7 Department of Orthopedics, Örebro University Hospital, Örebro, Sweden; 8 Capio Artro Clinic, FIFA Medical Centre of Excellence, Sophiahemmet Hospital, Stockholm, Sweden

**Keywords:** Anterior Cruciate Ligament, Epidemiology, Knee injuries, Sports medicine, Women in sport

## Abstract

**Objective:**

To determine and compare the incidence rate (IR) of anterior cruciate ligament reconstruction (ACL-R) among higher-level athletes across six sports in the Swedish National Knee Ligament Registry (SNKLR).

**Methods:**

Patient data from the SNKLR, between 2005 and 2020, was linked to team and event data of six sports (football, handball, basketball, ice hockey, floorball and alpine sports) to identify higher-level athletes aged 15–40 with ACL-R. Unadjusted and adjusted IR ratios (IRRs) with 99% CIs were calculated between sports, sex, age and divisions.

**Results:**

Female athletes had a 3.3 times higher ACL-R IR compared with males (1.08 vs 0.32, IRR=3.33, 99% CI: 2.65 to 4.19) per 1000 athlete exposures (AE). Basketball had the largest difference in ACL-R IR per 1000 AE between females and males (1.26 vs 0.22, IRR=5.69, 99% CI: 2.79 to 11.60). Female second-division athletes had higher ACL-R IR per 1000 AE compared with female highest-division athletes (1.27 vs 0.76, IRR=1.67, 99% CI: 1.30 to 2.15). No significant association between age and IR was observed. Compared with football, lower ACL-R IR was observed in floorball and ice hockey in females, as well as in floorball, basketball and ice hockey in males.

**Conclusion:**

Female athletes had higher ACL-R IRs than males and second-division female athletes had higher ACL-R IRs than highest-division female athletes. Lower ACL-R IRs were observed in floorball and ice hockey compared with football for both sexes. The remaining sports had ACL-R IRs similar to football, except basketball where rates were lower for male athletes.

**WHAT IS ALREADY KNOWN ON THIS TOPIC**

- While it is widely known that higher-level athletes face an elevated risk of sustaining an anterior cruciate ligament (ACL) injury and of undergoing ACL reconstruction (ACL-R), the precise extent of this heightened risk remains uncertain and the difference between females and males within the same cohorts over several seasons and with equal participation rates has been scarcely studied.

**WHAT THIS STUDY ADDS**

- This nationwide registry-based cohort study reports differences in ACL-R crude and adjusted incidence rates (IRs) between higher-level athletes across 15 years in different popular sports demonstrating comparable ACL-R IR across football, handball, basketball and alpine sports in females, as well as in football, handball and alpine sports in males.
- Female athletes had a 3.3 times higher ACL-R IR compared with their male counterparts and female athletes in the second division had a 1.7 times higher ACL-R IR than females in the highest division.

**HOW THIS STUDY MIGHT AFFECT RESEARCH, PRACTICE OR POLICY**

- To our knowledge, this is the first and only nationwide study investigating ACL-R incidence in higher-level athletes performed with a well-functioning, high-quality and reliable national registry that covers over 90% of all ACL-R procedures.
- This study improves our current understanding and knowledge regarding ACL-R incidence in athletes and may serve as a reference for future studies.
- Future research using national registries may follow the current study to help us better understand athlete injuries and surgeries as well as the problems these athletes face in recovery.
- More research is warranted to investigate the potential for further improvement of prevention programmes and their adherence. Additionally, efforts should be made to reduce the difference in ACL injury rates between female and male athletes.

## Introduction

 Anterior cruciate ligament (ACL) ruptures are potential career-ending injuries in high-performance athletes causing significant disability, psychological stress, loss of motivation and risk for poor patient-reported outcome measures.^1^,^3^ Many elite athletes experience problems on the way to recovery as well as during rehabilitation and at least one out of every five athletes does not return to sports.^4^ It is therefore vital to better understand the epidemiology of ACL injuries to be able to develop and improve effective prevention and treatment alternatives for athletes.^5^

Studies from different countries have presented the incidence of ACL injuries in elite and college athletes in various sports.^6^,^9^ However, variations in sporting exposure, climate conditions, turf material, healthcare systems as well as different methods of reporting ACL injury incidence make it difficult to compare ACL incidence rates (IRs) between studies.^8^ Nevertheless, there are two general concepts that seem to be consistent in almost all studies regardless of the country or location. The first is the higher IR of ACL injury in females compared with males when exposed to the same knee-strenuous sport with equal participation rates.^6 7 9^ The second general concept found in the literature is that professional and elite athletes have a higher likelihood of sustaining an ACL injury as well as having reconstructive surgery to continue with their sport.^8^ Although ACL injuries have been extensively studied, there are few studies comparing ACL injury incidence between sexes, divisions and different sports within the same cohorts and with equal sports participation over long periods of time.

The Swedish National Knee Ligament Registry (SNKLR) registers ACL reconstructions (ACL-R) with a high degree of coverage on a national level in Sweden as well as provides important information about the injury date, type of sport or activity during injury as well as information concerning concomitant injuries. Therefore, the SNKLR could be a useful tool to indirectly assess the ACL IR in athlete populations a with high likelihood of undergoing ACL-R. However, the registry does not provide information regarding the level of sports participation.^10 11^

The aim of this study was to investigate the incidence of ACL-R in higher-level athletes, defined in this study as athletes competing in the two highest divisions in knee-strenuous team sports in Sweden (football, handball, basketball, ice hockey, and floorball) as well as Swedish athletes competing in official International Ski and Snowboard Federation (FIS) tournaments. We aimed to find potential differences in IR of ACL-R between sexes, level of sports, age and sports participation by combining data from six sports organisations in Sweden and the SNKLR during the period of 2005–2020.

## Material and methods

### Study sample and eligibility criteria

This registry cohort study is the first part of the SWEREX study (SWEdish nationwide incidence and REturn to sports after ACL-R(X)). The SNKLR was cross-checked with the databases from six sports associations/federations (football, handball, basketball, floorball, ice hockey and alpine sports) with complete data regarding athlete participation in official matches or events between January 2005 and December 2020. Data regarding training sessions for the above-mentioned sports was not accessible for the current study. The linkage process was performed using athletes’ unique Swedish personal identity numbers or in cases this was not possible, by names, dates of birth and sex. All higher-level athletes in the ages of 15–40 years of age, competing in seasons with complete regular-season (all sports) and postseason (handball, basketball, ice hockey and floorball) match/event data were included in this study. The Swedish Ice Hockey Association could only provide data from the highest division for females, hence only first-division female ice hockey players were included. Athletes participating in seasons with incomplete data were excluded.

Complete data with the unique Swedish personal identity number and full match data was available from the Swedish Football Association for seasons 2009–2020, the Swedish Handball Federation for seasons 2012–2020, the Swedish Basketball Federation for seasons 2010–2019 and the Swedish Floorball Federation for seasons 2010–2020. Match data from the Swedish Ice hockey Association for seasons 2011–2019 and event data from the Swedish Ski Association and FIS for seasons 2005–2020 could not be provided with the Swedish personal identity number, hence individuals were identified by names, dates of birth and sex. ACL-R in higher-level athletes was defined as a primary or revision ACL-R with or without concomitant surgeries/reconstructions registered in the SNKLR from 2005 to 2020. The ACL injury leading to ACL-R was sustained by an athlete registered in one of the six sports associations/federations while participating in the same sport as that of the sports association/federation (information provided by the SNKLR). Also, the ACL injury had to be sustained within 3 months (before or after) of an official athlete event to ensure that the athlete was participating in the highest level when injured. If the date of ACL injury was missing, then the date of ACL-R was used.

This study was planned and performed according to the Strengthening the Reporting of Observational Studies in Epidemiology guidelines (STROBE)^12^ as well as reported according to the REporting of studies Conducted using Observational Routinely collected health Data (RECORD) Statement, an extension to the STROBE guidelines.^13^

### Outcome measurements and covariates

The primary outcomes for this study were the ACL-R IR per 1000 athlete exposures (AE) and the ACL-R IR per 1000 athlete seasons (AS). AE defined as one athlete participating in one official game or race, was used to better compare IR between different sports with different exposures. AS was used to evaluate the annual/seasonal incidence of ACL-R irrespective of exposure and was defined as one athlete participating in one season of sport (comparable with person-years). Variables used from the SNKLR were sex, date of injury, age at time of injury, side of injury and surgical data (type of surgery). The SNKLR does not use ICD codes or algorithms to identify variables.

### Equity, diversity and inclusion statement

No specific considerations were undertaken during the planning and design regarding equity, diversity and inclusion principles. However, our study population consisted of a specific group of higher-level athletes in the six above-mentioned sports regardless of ethnic origin, culture, educational level and socioeconomic background. The study was conducted in a single high-income country and from able-bodied sports. Sex was accounted for, and female and male athletes were analysed separately and compared. The author group consists of two women and four men with different backgrounds and with different ranges of experience clinically and in research (ie, three orthopaedic surgeons, one statistician, one physical therapist and one engineer).

### Statistical analyses

Statistical analyses were performed using IBM SPSS Statistics for Windows (V.29.0.2.0 Armonk, New York, USA: IBM). Data analysis and presentation were consistent with the CHecklist for statistical Assessment of Medical Papers statement.^14^ Categorical data in table 1 are presented with frequency and per cent and continuous data with mean values and SD.

**Table 1:** Exposure and ACL-R characteristics and incidence in different sports

Athlete characteristics		Football	Handball	Basketball	Ice hockey	Floorball	Alpine sports	Total
Exposure characteristics of all athletes								
Seasons	Males/females, n	12/12	9/9	10/10	9/9	11/11	16/16	
Athletes	Females, n (%)	3407 (50)	1541 (48)	1850 (51)	1044 (25)	4005 (55)	1180 (44)	13 027 (47)
	Males, n (%)	3394 (50)	1694 (52)	1748 (49)	3082 (75)	3256 (45)	1477 (56)	14 651 (53)
Athlete seasons (AS)	Females, n (%)	8815 (45)	4220 (45)	5023 (52)	2980 (24)	12 474 (55)	3551 (42)	37 063 (45)
	Males, n (%)	10 711 (55)	5160 (55)	4562 (48)	9509 (76)	10 281 (45)	4977 (58)	45 200 (55)
Athlete exposure (match/race)	Females, n (%)	133 504 (40)	66 039 (39)	71 068 (47)	62 820 (17)	197 059 (51)	64 116 (41)	594 606 (39)
	Males, n (%)	203 400 (60)	102 451 (61)	81 330 (53)	307 715 (83)	190 238 (49)	90 894 (59)	976 028 (61)
Age (years)	Females, mean (SD)	22.2 (4.4)	21.7 (3.5)	20.5 (4.4)	20.8 (4.0)	21.2 (3.9)	18.8 (2.7)	21.1 (4.1)
	Males, mean (SD)	24.6 (4.9)	23.1 (4.5)	22.5 (4.8)	23.8 (5.0)	23.4 (4.5)	19.7 (3.8)	23.2 (4.9)
Division*	Highest division							
	Females, n (%)	3331 (38)	2124 (45)	1448 (50)	2980 (41)	3426 (48)	–	13 309 (43)
	Males, n (%)	5416 (62)	2570 (55)	1444 (50)	4274 (59)	3665 (52)	–	17 369 (57)
	Second highest division							
	Females, n (%)	5484 (51)	2096 (45)	3575 (53)	–	9048 (58)	–	20 203 (47)
	Males, n (%)	5295 (49)	2590 (55)	3118 (47)	5235 (100)	6616 (42)	–	22 854 (53)
Discipline*	Alpine skiing							
	Females, n (%)	–	–	–	–	–	3175 (46)	3175 (46)
	Males, n (%)	–	–	–	–	–	3713 (54)	3713 (54)
	Freestyle skiing							
	Females, n (%)	–	–	–	–	–	376 (23)	376 (23)
	Males, n (%)	–	–	–	–	–	1264 (77)	1264 (77)
ACL-R characteristics								
ACL-R	Females, n (%)	178 (60)	98 (63)	88 (83)	5 (16)	175 (75)	60 (65)	604 (66)
	Males, n (%)	119 (40)	57 (37)	18 (17)	26 (84)	56 (25)	32 (35)	308 (34)
Age (years)	Females, mean (SD)	22.0 (4.1)	20.9 (3.0)	19.6 (3.9)	20.8 (3.8)	20.9 (3.3)	19.5 (3.0)	20.9 (3.6)
	Males, mean (SD)	25.0 (4.0)	22.2 (3.6)	23.3 (5.2)	24.3 (4.8)	23.2 (4.1)	20.3 (2.2)	23.5 (4.2)
Division	Highest division							
	Females, n (%)	64 (54)	44 (61)	25 (68)	5 (24)	60 (71)	–	198 (60)
	Males, n (%)	54 (46)	28 (39)	12 (32)	16 (76)	24 (29)	–	134 (40)
	Second highest division							
	Females, n (%)	114 (80)	54 (65)	63 (91)	–	115 (78)	–	346 (71)
	Males, n (%)	65 (20)	29 (35)	6 (9)	10 (100)	32 (22)	–	142 (29)
Discipline	Alpine skiing							
	Females, n (%)	–	–	–	–	–	54 (69)	54 (69)
	Males, n (%)	–	–	–	–	–	24 (31)	24 (31)
	Freestyle skiing							
	Females, n (%)	–	–	–	–	–	6 (43)	6 (43)
	Males, n (%)	–	–	–	–	–	8 (57)	8 (57)
Type of operation	Primary ACL-R, n (%)	224 (75)	127 (82)	85 (80)	25 (81)	192 (83)	72 (78)	725 (80)
	Contralateral ACL-R, n (%)	34 (12)	14 (9)	11 (10)	1 (3)	21 (9)	12 (13)	93 (10)
	Revision ACL-R, n (%)	39 (13)	14 (9)	10 (10)	5 (16)	18 (8)	8 (9)	94 (10)

* Athlete seasons (AS).

ACL-R, anterior cruciate ligament reconstruction.

ACL-R IR and IR ratios (IRR) were calculated with negative binomial regression with a log link function and an offset variable to account for different exposure times. Negative binomial regression is used as in this case when count data exhibit overdispersion. An extra parameter is introduced in the models to account for the overdispersion using a maximum likelihood estimate. Match exposure was calculated by the ‘athlete-participation method’ described by Stovitz and Shier.^15^ The natural logarithm of AE and AS were entered as offset variables in the models. ACL-R IR was presented as the number of ACL-R per 1000 AE or per 1000 AS with a 99% Wald CI. All count data was aggregated to the team seasonal level before analysis, except for alpine sports that were aggregated to the seasonal level as alpine sports are individual-based rather than team-based sports. Age at the time of injury and division were entered as covariates in the adjusted regression models.

All analyses were two-sided and the significance level was set at p<0.01.

## Results

### Exposure characteristics

As shown in table 1, a total of 27 678 athletes were included in the study giving rise to a total of 82 263 AS with a mean age (SD) of 22.3 (4.7) at the time of injury. In this cohort, 13 027 (47%) female athletes with a total of 37 063 (45%) AS and 14 651 (53%) male athletes with 45 200 (55%) AS were included. 43% (n=30 678) played in the highest divisions. In alpine sports, 81% (n=6888) of the athletes competed in alpine skiing compared with 19% (n=1640) in freestyle skiing. A total of 1 570 634 AE for the whole cohort were identified.

### ACL-R characteristics and surgical type

A total of 912 ACL-R in 828 athletes were identified and met the inclusion criteria in the present cohort. Additionally, we found 17 athletes (2 in handball, 5 in football, 5 in basketball, 4 in floorball and 1 in alpine skiing) with ACL-R that were injured in a sport other than that of their sports association/federation, thereby not being included in the IR analysis in the current study. The mean age (SD) at the time of injury was 21.6 (4.0) years. 60% (n=488) were active in the second-highest division and 66% (n=604) were female athletes in the current study. Most of the cases were primary ACL-R surgery, 80% (n=725), while 10% (n=93) were contralateral ACL-R surgery and 10% (n=94) were ACL-R revision surgery.

### ACL-R incidence by sex

The unadjusted and adjusted ACL-R IR per 1000 AE and per 1000 AS with IRR per sex are presented in table 2. Adjusting for age and division only slightly altered the overall analysis, hence we focus on describing only the adjusted results. Female athletes had a 3.3 times higher ACL-R IR per 1000 AE compared with males (1.08 vs 0.32, IRR=3.33, 99% CI: 2.65 to 4.19). In contrast, the IR of ACL-R per 1000 AS for female athletes was slightly lower and was 2.6 times higher than for male athletes (17.16 vs 6.58, IRR=2.61, 99% CI: 2.12 to 3.21), showing the difference of IR calculations between the two methods AE and AS. In percentage, the ACL-IR per season (%) for females was 1.72% and males 0.66%.

**Table 2:** ACL-R incidence rate per 1000 athlete exposures and per 1000 athlete seasons with incidence rate ratio per sex (99% CI)

		Football	Handball	Basketball	Ice hockey	Floorball	Alpine sports	Total
ACL-R incidence rate and incidence rate ratio per sex per 1000 athlete exposure								
Unadjusted IR	Female	1.33	1.48	1.24	0.08	0.89	0.99	1.05
(99% CI)		(1.10 to 1.62)	(1.14 to 1.93)	(0.93 to 1.65)	(0.02 to 0.26)	(0.73 to 1.09)	(0.62 to 1.59)	(0.93 to 1.18)
	Male (ref)	0.59	0.56	0.22	0.08	0.29	0.38	0.33
		(0.46 to 0.74)	(0.40 to 0.78)	(0.12 to 0.41)	(0.05 to 0.14)	(0.21 to 0.42)	(0.22 to 0.68)	(0.28 to 0.38)
Unadjusted IRR		2.28	2.67	5.61	0.94	3.02	2.59	3.21
(99% CI)		(1.68 to 3.10)	(1.73 to 4.10)	(2.86 to 11.00)	(0.26 to 3.36)	(2.02 to 4.52)	(1.27 to 5.29)	(2.63 to 3.91)
P value		<0.001	<0.001	<0.001	0.898	<0.001	<0.001	<0.001
Adjusted IR	Female	1.22	1.43	1.26	0.06	0.85	1.62	1.08
(99% CI)		(0.94 to 1.58)	(1.05 to 1.93)	(0.93 to 1.70)	(0.01 to 0.30)	(0.67 to 1.08)	(0.96 to 2.73)	(0.95 to 1.23)
	Male (ref)	0.63	0.58	0.22	0.09	0.32	0.53	0.32
		(0.48 to 0.82)	(0.41 to 0.83)	(0.12 to 0.41)	(0.04 to 0.20)	(0.22 to 0.46)	(0.31 to 0.89)	(0.27 to 0.38)
Adjusted IRR		1.95	2.44	5.69	0.62	2.68	3.08	3.33
(99% CI)		(1.27 to 3.02)	(1.49 to 4.01)	(2.79 to 11.60)	(0.07 to 5.20)	(1.65 to 4.33)	(1.58 to 6.03)	(2.65 to 4.19)
P value		<0.001	<0.001	<0.001	0.560	<0.001	<0.001	<0.001
ACL-R incidence rate and incidence rate ratio per sex per 1000 athlete seasons								
Unadjusted IR	Female	20.19	23.22	17.50	1.68	14.02	17.05	16.30
(99% CI)		(16.65 to 24.49)	(17.89 to 30.14)	(13.25 to 23.11)	(0.52 to 5.35)	(11.47 to 17.15)	(11.49 to 25.31)	(14.67 to 18.10)
	Male (ref)	11.11	11.05	3.95	2.73	5.45	6.41	6.81
		(8.77 to 14.07)	(7.85 to 15.54)	(2.15 to 7.25)	(1.63 to 4.58)	(3.84 to 7.72)	(3.91 to 10.49)	(5.88 to 7.89)
Unadjusted IRR		1.82	2.10	4.43	0.61	2.58	2.66	2.39
(99% CI)		(1.34 to 2.47)	(1.37 to 3.23)	(2.27 to 8.66)	(0.17 to 2.18)	(1.72 to 3.85)	(1.41 to 5.01)	(2.00 to 2.86)
P value		<0.001	<0.001	<0.001	0.321	<0.001	<0.001	<0.001
Adjusted IR	Female	19.45	22.51	17.92	1.28	13.47	18.39	17.16
(99% CI)		(15.03 to 25.17)	(16.71 to 30.32)	(13.31 to 24.12)	(0.24 to 6.79)	(10.63 to 17.07)	(10.84 to 31.21)	(15.23 to 19.34)
	Male (ref)	11.39	11.22	3.84	2.94	5.59	6.26	6.58
		(8.67 to 14.97)	(7.88 to 15.95)	(2.06 to 7.15)	(1.39 to 6.21)	(3.85 to 8.14)	(3.70 to 10.60)	(5.64 to 7.67)
Adjusted IRR		1.71	2.01	4.67	0.44	2.41	2.94	2.61
(99% CI)		(1.11 to 2.63)	(1.23 to 3.27)	(2.30 to 9.48)	(0.05 to 3.72)	(1.49 to 3.89)	(1.48 to 5.84)	(2.12 to 3.21)
P value		0.001	<0.001	<0.001	0.318	<0.001	<0.001	<0.001

Statistical method: Negative binomial regression using maximum likelihood estimation, with log link function.

Covariates in adjusted regression model: age, division.

ACL-R, anterior cruciate ligament reconstruction; IR, incidence rate; IRR, incidence rate ratio.

**Figure 1:**
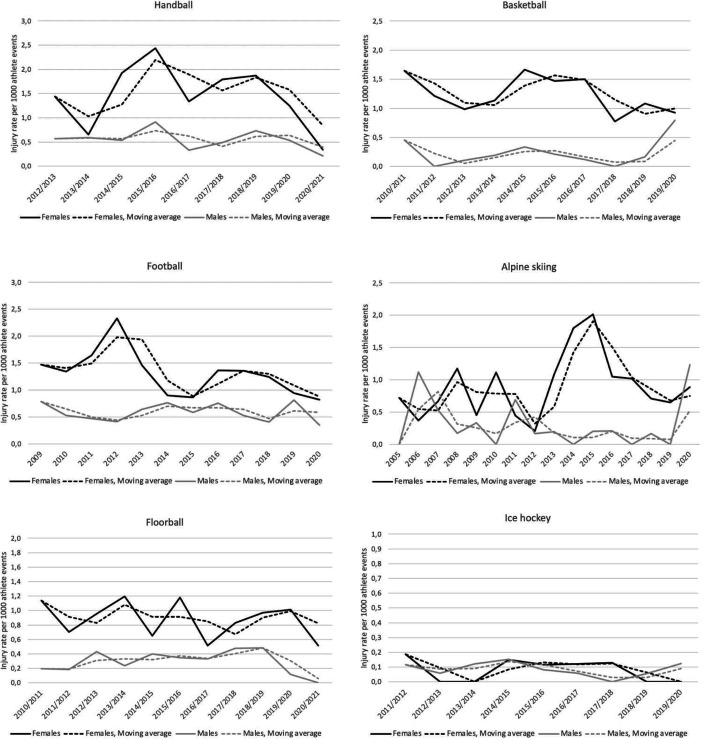
Seasonal trend for the unadjusted ACL-R incidence rates in female and male higher-level athletes in six different sports from 2005 to 2020.

The greatest difference in incidence between females and males was seen in basketball where female athletes had a 5.7 times higher ACL-R per 1000 AE (1.26 vs 0.22, IRR=5.69, 99% CI: 2.79 to 11.60) and a 4.7 times higher ACL-R IR per 1000 AS (17.92 vs 3.84, IRR=4.67, 99% CI: 2.30 to 9.48) compared with males. In contrast, no clear difference in ACL-R IR per 1000 AE (0.06 vs 0.09, IRR=0.62, 99% CI: 0.07 to 5.20) and per 1000 AS (1.28 vs 2.94, IRR=0.44, 99% CI: 0.05 to 3.72) was observed in ice hockey. To better understand and visualise the factual variation of ACL-R in each sport throughout various seasons, we present figure 1 with unadjusted ACL-R IR per 1000 AE variations within the different sports per season including moving average .

### ACL-R incidence by division and age

Table 3 outlines the unadjusted and adjusted comparison between divisions and skiing disciplines for females and males separately. Analysing each sport separately, we could not see any differences between divisions in females or males. Nevertheless, there was a general tendency of higher ACL-R IR per 1000 AE in female second-division athletes compared with first-division athletes, and when pooling team sports together we saw that second-division female athletes had a 1.7 times higher adjusted ACL-R IR per 1000 AE compared with first division athletes (1.27 vs 0.76, IRR=1.67, 99% CI: 1.30 to 2.15). However, this tendency was not seen in male sports. In male alpine sports, freestyle skiing had a higher adjusted ACL-R IR per 1000 AE than alpine skiing (1.07 vs 0.29, IRR=3.63, 99% CI: 1.12 to 11.77). Conversely, we saw no difference between alpine sports disciplines in females.

**Table 3:** ACL-R incidence rate per 1000 athlete exposures with incidence rate ratio per divisions by sex (99% CI)

		Football	Handball	Basketball	Ice hockey	Floorball	Alpine sports	Total
ACL-R incidence rate and incidence rate ratio per division per 1000 athlete exposure in females								
Unadjusted IR	Second highest division/freestyle skiing	1.41	1.89	1.40	–	0.95	2.30	1.26
(99% CI)		(1.11 to 1.80)	(1.32 to 2.70)	(1.01 to 1.93)		(0.74 to 1.21)	(0.80 to 6.58)	(1.09 to 1.46)*
	Highest division/alpine skiing (ref)	1.21	1.17	0.96	0.08	0.79	0.88	0.79
		(0.88 to 1.67)	(0.79 to 1.74)	(0.58 to 1.61)	(0.03 to 0.25)	(0.57 to 1.11)	(0.62 to 1.25)	(0.65 to 0.96)*
Unadjusted IRR		1.16	1.61	1.45	–	1.19	2.62	1.59
(99% CI)		(0.78 to 1.74)	(0.94 to 2.75)	(0.79 to 2.66)		(0.79 to 1.81)	(0.86 to 7.93)	(1.25 to 2.03)*
P value		0.328	0.022	0.117		0.275	0.025	<0.001
Adjusted IR	Second highest division/freestyle skiing	1.36	1.84	1.41	–	0.9	2.13	1.27
(99% CI)		(1.03 to 1.79)	(1.27 to 2.65)	(1.00 to 1.99)		(0.70 to 1.17)	(0.72 to 6.30)	(1.10 to 1.47)*
	Highest division/alpine skiing (ref)	1.28	1.17	0.95	0.08	0.87	1.04	0.76
		(0.89 to 1.85)	(0.79 to 1.74)	(0.54 to 1.69)	(0.03 to 0.26)	(0.60 to 1.25)	(0.64 to 1.69)	(0.63 to 0.93)*
Adjusted IRR		1.06	1.57	1.48	–	1.04	2.05	1.67
(99% CI)		(0.64 to 1.76)	(0.92 to 2.68)	(0.73 to 3.02)		(0.65 to 1.68)	(0.58 to 7.27)	(1.30 to 2.15)*
P value		0.772	0.031	0.157		0.820	0.145	<0.001
ACL-R incidence rate and incidence rate ratio per division per 1000 athlete exposure in males								
Unadjusted IR	Second highest division/freestyle skiing	0.64	0.70	0.14	0.06	0.32	1.10	0.32
(99% CI)		(0.47 to 0.89)	(0.40 to 1.22)	(0.05 to 0.39)	(0.03 to 0.15)	(0.20 to 0.50)	(0.36 to 3.39)	(0.26 to 0.40)*
	Highest division/alpine skiing (ref)	0.53	0.46	0.33	0.11	0.27	0.30	0.31
		(0.37 to 0.75)	(0.26 to 0.80)	(0.15 to 0.69)	(0.05 to 0.21)	(0.16 to 0.46)	(0.14 to 0.67)	(0.24 to 0.38)*
Unadjusted IRR		1.22	1.53	0.42	0.59	1.17	3.65	1.04
(99% CI)		(0.76 to 1.96)	(0.70 to 3.35)	(0.11 to 1.53)	(0.20 to 1.73)	(0.58 to 2.34)	(0.92 to 14.48)	(0.76 to 1.42)*
P value		0.291	0.164	0.083	0.209	0.566	0.016	0.727
Adjusted IR	Second highest division/freestyle skiing	0.62	0.71	0.14	0.06	0.32	1.07	0.35
(99% CI)		(0.44 to 0.87)	(0.41 to 1.23)	(0.05 to 0.41)	(0.03 to 0.15)	(0.20 to 0.51)	(0.40 to 2.85)	(0.27 to 0.44)*
	Highest division/alpine skiing (ref)	0.55	0.46	0.28	0.11	0.26	0.29	0.30
		(0.38 to 0.78)	(0.26 to 0.80)	(0.11 to 0.74)	(0.05 to 0.24)	(0.15 to 0.45)	(0.14 to 0.59)	(0.23 to 0.40)*
Adjusted IRR		1.13	1.54	0.51	0.57	1.24	3.63	1.15
(99% CI)		(0.68 to 1.88)	(0.70 to 3.42)	(0.12 to 2.20)	(0.16 to 2.06)	(0.59 to 2.61)	(1.12 to 11.77)	(0.79 to 1.67)*
P value		0.532	0.160	0.232	0.261	0.458	0.005	0.350

Statistical method: Negative binomial regression using maximum likelihood estimation, with log link function.

Covariates in adjusted regression model: age.

* Not including alpine sports.

ACL-R, anterior cruciate ligament reconstruction; IR, incidence rate; IRR, incidence rate ratio.

The analysis did not provide strong evidence of an association between age and ACL-R IR per 1000 AE as the result was not statistically significant in either females or males in any of the described sports, irrespective of unadjusted or adjusted analyses, shown in table 4.

**Table 4:** ACL-R incidence rate ratio of age per 1000 athlete exposures by sex (99% CI)

		Football	Handball	Basketball	Ice hockey	Floorball	Alpine sports	Total
Female	**Unadjusted IRR**	0.94	0.88	0.97	0.91	0.92	1.38	0.99
	(**99% CI**)	(0.82 to 1.07)	(0.70 to 1.11)	(0.87 to 1.08)	(0.40 to 2.07)	(0.81 to 1.03)	(0.92 to 2.07)	(0.93 to 1.05)
	**P value**	0.222	0.150	0.451	0.770	0.061	0.039	0.534
	**Adjusted IRR**	0.95	0.90	1.01	0.91	0.92	1.24	1.02
	(**99% CI**)	(0.81 to 1.12)	(0.72 to 1.13)	(0.89 to 1.14)	(0.40 to 2.07)	(0.80 to 1.06)	(0.79 to 1.93)	(0.96 to 1.08)
	**P value**	0.431	0.219	0.885	0.770	0.128	0.224	0.423
Male	**Unadjusted IRR**	0.89	1.00	1.16	1.12	1.03	1.22	1.03
	(**99% CI**)	(0.70 to 1.12)	(0.75 to 1.32)	(0.88 to 1.52)	(0.69 to 1.80)	(0.81 to 1.30)	(0.54 to 2.72)	(0.94 to 1.14)
	**P value**	0.175	0.966	0.166	0.551	0.751	0.532	0.385
	**Adjusted IRR**	0.90	1.02	1.09	0.97	1.06	1.00	1.04
	(**99% CI**)	(0.71 to 1.16)	(0.77 to 1.35)	(0.78 to 1.51)	(0.54 to 1.73)	(0.82 to 1.36)	(0.53 to 1.86)	(0.94 to 1.15)
	**P value**	0.294	0.844	0.524	0.887	0.569	0.987	0.291

Statistical method: Negative binomial regression using maximum likelihood estimation, with log link function.

Covariates in adjusted regression model: division.

ACL-R, anterior cruciate ligament reconstruction; IRR, incidence rate ratio.

### ACL-R incidence by sports

The unadjusted and adjusted IRR between all sports compared with football is shown per 1000 AE and per 1000 AS in table 5 for females and for males. There were no differences between adjusted ACL-R IR per 1000 AE nor per 1000 AS in female handball, basketball and alpine sports compared with female football. Female floorball had lower adjusted ACL-R IR per 1000 AE (0.85 vs 1.22, IRR=0.64, 99% CI: 0.48 to 0.86) and adjusted ACL-R IR per 1000 AS (13.47 vs 19.45, IRR=0.68, 99% CI: 0.51 to 0.91) compared with football. The lowest ACL-R IR in female sports compared with football was seen in ice hockey for both IR per 1000 AE (0.06 vs 1.22, IRR=0.07, 99% CI: 0.02 to 0.22) and per 1000 AS (1.28 vs 19.45, IRR=0.08, 99% CI: 0.02 to 0.26). In contrast, when comparing male football-adjusted ACL-R IR per 1000 AE and per 1000 AS with other sports, only handball and alpine sports showed no difference. Male floorball showed a 0.5 times lower IR per 1000 AE and per 1000 AS compared with football. Following floorball, male basketball had 0.4-fold lower IR per 1000 AE and per 1000 AS. Once again, ice hockey had the lowest adjusted ACL-R IR compared with football with an IRR per 1000 AE at 0.15 (0.09 vs 0.63, IRR=0.15, 99% CI: 0.08 to 0.27) and an IRR per 1000 AS at 0.26 (2.94 vs 11.39, IRR=0.26, 99% CI: 0.14 to 0.46).

**Table 5:** ACL-R incidence rate ratio per 1000 athlete exposures and per 1000 athlete seasons, comparison of sports in females and males with respect to football (99% CI)

ACL-R incidence rate ratio per 1000 athlete exposure								
	Females				Males			
	Unadjusted		Adjusted		Unadjusted		Adjusted	
	IRR (99% CI)	P value	IRR (99% CI)	P value	IRR (99% CI)	P value	IRR (99% CI)	P value
Football	1 (ref)		1 (ref)		1 (ref)		1 (ref)	
Handball	1.11 (0.80 to 1.55)	0.405	1.14 (0.82 to 1.60)	0.304	0.96 (0.61 to 1.49)	0.798	1.03 (0.64 to 1.66)	0.884
Basketball	0.93 (0.66 to 1.30)	0.575	0.88 (0.61 to 1.26)	0.358	0.38 (0.19 to 0.74)	<0.001	0.40 (0.20 to 0.80)	<0.001
Ice hockey	0.06 (0.02 to 0.19)	<0.001	0.07 (0.02 to 0.22)	<0.001	0.14 (0.08 to 0.26)	<0.001	0.15 (0.08 to 0.27)	<0.001
Floorball	0.67 (0.50 to 0.88)	<0.001	0.64 (0.48 to 0.86)	<0.001	0.51 (0.32 to 0.79)	<0.001	0.53 (0.33 to 0.84)	<0.001
Alpine sports	0.71 (0.47 to 1.09)	0.040	0.74 (0.43 to 1.27)	0.151	0.72 (0.35 to 1.47)	0.235	0.93 (0.36 to 2.42)	0.851

ACL-R incidence rate ratio per 1000 athlete seasons								
	Females				Males			
	Unadjusted		Adjusted		Unadjusted		Adjusted	
	IRR (99% CI)	P value	IRR (99% CI)	P value	IRR (99% CI)	P value	IRR (99% CI)	P value
Football	1 (ref)		1 (ref)		1 (ref)		1 (ref)	
Handball	1.15 (0.83 to 1.60)	0.272	1.13 (0.81 to 1.58)	0.340	0.99 (0.64 to 1.54)	0.972	1.07 (0.67 to 1.70)	0.724
Basketball	0.87 (0.62 to 1.22)	0.278	0.84 (0.59 to 1.21)	0.220	0.36 (0.18 to 0.69)	<0.001	0.40 (0.20 to 0.79)	<0.001
Ice hockey	0.08 (0.03 to 0.27)	<0.001	0.08 (0.02 to 0.26)	<0.001	0.25 (0.14 to 0.44)	<0.001	0.26 (0.14 to 0.46)	<0.001
Floorball	0.69 (0.53 to 0.92)	<0.001	0.68 (0.51 to 0.91)	<0.001	0.49 (0.32 to 0.76)	<0.001	0.53 (0.34 to 0.84)	<0.001
Alpine sports	0.84 (0.56 to 1.26)	0.271	0.77 (0.46 to 1.28)	0.183	0.57 (0.31 to 1.06)	0.020	0.72 (0.30 to 1.71)	0.327

Statistical method: Negative binomial regression using maximum likelihood estimation, with log link function.

Covariates in adjusted regression model: age.

ACL-R, anterior cruciate ligament reconstruction; IRR, incidence rate ratio.

## Discussion

The main findings of the current study are the different ACL-R IR in the six studied sports, a higher ACL-R IR in female athletes compared with male athletes and a tendency for higher IR for second-division female athletes compared with first-division. This study also presents the differences in ACL-R IR between five different sports compared with football. In both females and males, lower ACL-R IR were observed in floorball and ice hockey while handball and alpine skiing showed comparable IR. Basketball differed from the rest of sports by exhibiting ACL-R IR in females comparable to football but lower ACL-IR in males. These findings provide important epidemiological information on where our prevention strategies for ACL injuries in athletes should be directed. They also help pinpoint priorities for future research, practice improvements and policy initiatives within sports organisations.

### Variations between sexes, divisions, age and sports

Female athletes in our cohort had 3.3 times higher IR of ACL-R per 1000 AE compared with male athletes. This is in line with previous findings in which female athletes in high-risk sports such as football and basketball had 2.6–3.6 times higher incidence of ACL tears.^6 7 9^ However, these studies include various sporting groups (elite, professional, college, high school and amateur athletes) and only a few epidemiological studies have reported ACL injury rates in elite athletes in both sexes during the same period with an equal number of exposures and with the same methods and none have adjusted for age and division.

With the exception of ice hockey, our study presents a high number of exposures in each sport and similar participation rates between males and females, thereby demonstrating differences in IR between males and females not only in football and basketball but also in handball, floorball and alpine skiing.

Surprisingly, we saw a 5.7 times higher IR per 1000 AE and 4.7 times higher ACL-R IR per 1000 AS in female basketball players compared with males mainly due to very low ACL-R IR in male basketball players. Prodromos *et al* also found a high female–male ratio of ACL injuries in basketball and saw that it differed between different levels of competition with a female–male ACL injury ratio at 0.95 in professional athletes, 3.63 in college students and 4.4 in high school students.^7^ Our findings could be due to a higher degree of adherence to specific Swedish injury prevention programmes (Basketsmart) by male athletes compared with female athletes.^16^ However, it is important to mention that the degree of adherence to this prevention programme is unknown. We can only hypothesise whether this marked difference in IRR of ACL-R in basketball compared with other sports is due to a difference in the level of competition between female and male basketball players or because of better adherence to prevention programmes by male basketball athletes and further studies analysing these findings are warranted.

Exploring further the distinctions between divisions, there was a higher IR of ACL-R in the second-highest division compared with the highest division when pooling all female sports together. However, we saw no differences between divisions in male sports. Interestingly, Beynnon *et al* saw a significantly higher non-contact ACL injury incidence in college athletes compared with high-school athletes when comparing the level of competition.^17^ Still, there are no former studies analysing differences in ACL and/or ACL-R IR between divisions, so we can only speculate to reason for our findings. This could to some extent be related to the degree of professionality athletes might have at the highest level compared with the second highest level. The teams in the second highest division in female sports might have less access to good training facilities and to medical teams helping with the awareness, preparation and prevention of ACL injuries compared with the higher divisions.

In skiing, we saw a difference in males between alpine downhill skiing and freestyle. This is not surprising since there are more acrobatic movements and jumps in freestyle skiing which should increase the risk of retaining an ACL injury. Our findings are supported by a recent systematic review and meta-analysis studying injury incidence in professional snow sports by Fu *et al* in which they found freestyle skiing to be the sport discipline with the highest overall injury incidence.^18^

Our study failed to demonstrate any important difference regarding age at the time of injury and ACL-R IR. The significance of age and ACL injury/ACL-R incidence is still poorly understood and most probably due to multifactorial factors.^19 20^ A tendency of higher incidence in younger athletes has formerly been reported and could be related to lower technical skills, poor decision making and physical capabilities during play.^20^ However, we could not confirm this in our cohort and further research may be needed.

The current study could indicate that sports that involve high-impact landings and contact such as football, handball and female basketball are particularly high-risk sports. This may be attributed to one-legged landings, typical for these sports and being a high-risk situation for knee valgus collapse leading to ACL injuries in both females and males.^21^,^23^ Our findings are consistent with previous studies that have reported the highest incidence of ACL injuries in high-impact landing and contact sports.^9^ Lower rates of ACL-R were observed in floorball which also involves high-risk situations such as sudden directional changes during rapid deceleration, however, with fewer jumping or one-legged landing situations.^24^ Another high-risk sport was alpine sports where ACL injuries frequently occur during falls or loss of control with forces applied by the ski and snow causing the ‘slip-catch’ mechanism, differing this sport from the rest.^25^ Prevention strategies should continue to be directed toward improving neuromuscular control in these high-risk situations.

It is not surprising that ice hockey is the sport with the lowest IR of ACL-R in both female and male athletes. In accordance with our results, previous studies have not been able to show any differences in IR of ACL tears between males and females in ice hockey.^9^ The reason for this may be explained by the fact that ACL injuries in ice hockey predominately occur by contact mechanisms such as on-ice collision with another player or goalpost,^26^ a mechanism that may present a similar risk for ACL injuries in both female and male athletes. However, it could also be because we failed to achieve equal participation rates for both males and females in this sport.

Interestingly, we found very little difference when adjusting our analysis by age and division as covariates. This is probably because we had complete cohorts for all the included divisions thereby decreasing the need for adjustment. However, we found it important to address this important issue and clarify this by presenting both crude and adjusted values. We also saw very little difference when comparing sex and sports with our two alternative methods (per 1000 AE and per 1000 AS). The two methods were used to allow future comparison with other studies because of the varied methods of reporting ACL incidence in the current literature.^8^

The practicality of using AS is that it is easily comparable with person-years, helping us better understand the difference in ACL-R IR in higher-level athletes compared with previous epidemiological studies. For example, the incidence of ACL-R in Sweden for 20–39 year olds has been estimated to be 71 per 100 000 person-years (equal to 0.71 per 1000 person-years) by Granan *et al* when looking at the baseline epidemiology of the Scandinavian ACL registries.^11^ Compared with the total ACL-IR per 1000 AS in our study, this could arbitrarily mean that female athletes in our study could have 24 times higher ACL-R IR compared with the general population and that males have nine times higher ACL-R IR compared with the general population. Moses *et al* hypothesised that this could be explained by the fact that professional and elite athletes are more likely to have an ACL injury diagnosed, will most probably undergo surgery to continue to perform in their respective sport, have higher-level of play and may imply a greater force on the knee joint when changing direction or landing from jumps and finally professional and elite athletes have higher exposure annually.^8^ However, these calculations should be performed more systematically by comparing sport by sport to the general population excluding the athletes accounted for in our cohort and during the same period. Although of interest, we decided to focus on the comparison of differences between sex, division, age and sports and not on the comparison with the general population.

### Strengths and limitations

This large retrospective cohort study analyses the national incidence of ACL-R in higher-level athletes by cross-checking sport association data and a high-quality national health registry. The SNKLR is a well-functioning, high-quality and reliable national registry that covers over 90% of all ACL-R procedures in Sweden. The registry has included 52 734 primary ACL-R and 4040 revision ACL-R from the beginning of 2005 until the end of 2020.^10^ Given the fact that ACL-R is the preferred treatment of ACL injuries in elite athletes, we believe that the use of the SNKLR permits us to find almost all ACL injuries within these sports, thereby making it an important tool for assessing the incidence of ACL injuries indirectly. Various studies looking at IR of ACL injuries or return to sports rates after ACL-R in elite athletes report that more than 95% of these top-level athletes with ACL tears go through with ACL-R, thereby confirming the high likelihood for surgical reconstruction in this specific population after ACL injuries.^27^,^29^

However, there are several important limitations to this study. First, by using the SNKLR to identify individuals with ACL-R, we cannot be certain that we identify all individuals with ACL injuries. Furthermore, the absence of training exposure data is a significant limitation as the lack of training information does not permit us to present the total exposure and the total IRs. Another weakness is the fact that we did not have total minutes of playing time for all athletes in all sports. Ideally, the optimal method for calculating AE and IR of injuries in sports is by using individual-level exposure time. We therefore used the ‘Athlete Participation Method’ described by Stovitz and Shier when calculating our IR of ACL-R. This method may overestimate total AE time at risk and hence underestimate injury rates if studies are performed using team rosters only. However, we had exact data on which athletes participated in official matches/events, thereby minimising this risk. We also wanted to use a method that would allow us to compare IR between sports more arbitrarily since there are important differences in playing and competition time between sports. Another possible method is to use the ‘Athletes at Risk Method’ to estimate the team-level exposure time. This method may underestimate the exposure time if games go into overtime in the playoffs or postseason matches which we had in basketball, handball, floorball and ice hockey.^13^

Finally, potential confounding factors including differences in access to training or medical resources, prevalence of medical conditions, anatomical risk factors and generalised joint hypermobility as well as sport-specific playing conditions may influence our findings. These are factors known to affect the incidence of ACL injuries and cannot be accounted for in a study with our design. We therefore analysed our count data aggregated to team seasonal level in team sports and to seasonal level in alpine sports as we lacked plenty of individual data that could condition the ACL-R IR. These limitations are important for the readers to consider when interpreting the results.

## Conclusion

This nationwide registry-based cohort study demonstrates that female higher-level athletes were 3.3 times more likely to have an ACL-R compared with male athletes and that second-division female athletes had 1.7 higher ACL-R IR compared with first division athletes. The present study also highlights sport-specific differences in ACL-R IR with lower rates in floorball and ice hockey compared with football for both sexes. Handball and alpine skiing had similar ACL-R IRs to football while basketball showed comparable IR in females but lower IR in males. Finally, the results of the current study increase the understanding of the epidemiology of ACL-R in different sports in higher-level athletes as well as sex differences and can serve as a base for future studies looking at preventive measures. Future research should focus on improving the prevention of ACL injuries in these high-risk individuals as well as how to increase implementation and adherence of validated injury prevention programmes. Sports organisations should continue to promote and establish policies focused on injury prevention, ensuring they are accessible and consistently implemented across all levels.

## Data availability statement

No data are available.
